# New H6 influenza virus reassortment strains isolated from Anser fabalis in Anhui Province, China

**DOI:** 10.1186/s12985-017-0680-1

**Published:** 2017-02-21

**Authors:** Ye Ge, Hongliang Chai, Zhiqiang Fan, Xianfu Wang, Qiucheng Yao, Jian Ma, Si Chen, Yuping Hua, Guohua Deng, Hualan Chen

**Affiliations:** 1 State Key Laboratory of Veterinary Biotechnology, Harbin Veterinary Research Institute of Chinese Academy of Agricultural Sciences, Harbin, China; 2 College of Wildlife Resources, Northeast Forestry University, Harbin, Heilongjiang Province China; 3 School of Life Sciences, Anqing Normal University, Anqing, Anhui Province China; 4 Natural Protection & Management Station of Forestry Department Centre of Anhui Province, Hefei, Anhui Province China

**Keywords:** Avian influenza virus, Anser fabalis, H6 subtype

## Abstract

**Background:**

H6 subtype avian influenza viruses are globally distributed and, in recent years, have been isolated with increasing frequency from both domestic and wild bird species as well as infected humans. Many reports have examined the viruses in the context of poultry or several wild bird species, but there is less information regarding their presence in migratory birds.

**Methods:**

Hemagglutination and hemagglutination inhibition tests were used to measure HA activity for different HA subtypes. Whole viral genomes were sequenced and analysed using DNAstar and MEGA 6 to understand their genetic evolution. Pathogenicity was evaluated using a mouse infection model.

**Results:**

We isolated 13 strains of H6 virus from faecal samples of migratory waterfowl in Anhui Province of China in 2014. Phylogenetic analysis showed gene reassortment between Eurasian and North American lineages. Five of the identified H6 strains had the ability to infect mice without adaptation.

**Conclusion:**

Our findings suggest that regular surveillance of wild birds, especially migratory birds, is important for providing early warning and control of avian influenza outbreaks.

## Background

Avian influenza virus (AIV) is an important zoonotic pathogen [1] and can be classified into 16 hemagglutinin (HA) subtypes and 9 neuraminidase (NA) subtypes based on the antigenicity of these two surface glycoproteins. At least 136 species have been discovered in 26 different families of wild birds and harbour 144 subtypes of influenza A virus [1–4]. Most subtypes are found not only in common aquatic birds but also in lower mammals, such as bats, as has been demonstrated in recent reports [1, 5–7]. In Qinghai Lake, China in 2005, massive numbers of migrating birds died by H5N1, and this virus subsequently spread worldwide [8, 9]. In recent years, different influenza virus subtypes (such as H5, H6, H7, and H10) have emerged in succession in poultry, crossed the species barrier and caused frequent outbreaks of human infection [1, 10–15]. The continuous outbreaks of avian influenza in recent decades have alarmed and increased focus on the role of wild birds, as major reservoirs, that maintain the persistence and variation of AIV, facilitating viral spread and disease occurrence.

H6 AIV was first isolated from turkeys in Massachusetts, USA in 1965 [10, 16]. Currently, H6 AIVs has a worldwide distribution, and strains of the virus have been detected in various animal species [17]. On June 21, 2013, the first case of a human infection by an avian-origin H6N1 influenza A virus (A/Taiwan/2/2013, Taiwan2) was reported by the Taiwan Centres for Disease Control [11, 18]. Human infection with H6 AIVs in mainland China has not been reported, but a serum antibody positive for the H6 virus was found in poultry workers [19]. More than 30% of the H6 AIVs circulating in poultry in China have enhanced affinity to human-like receptors (ɑ-2, 6 NeuAcGal). Some H6 subtypes can also infect mice without prior adaptation, and some can be efficiently transmitted among guinea pigs [17]. These events indicate that H6 AIV can cross species barriers and directly infect humans. As the natural host of AIVs, wild birds play an important role in virus reassortment as well as intra- and interspecies transmission [4, 5]. However, there is little research regarding the biological properties of H6 AIV strains from wild birds and their potential threat to mammals compared to the many studies of H6 AIV in poultry.

In the present study, we isolated 13 H6 AIV strains after surveying wild birds in the wetlands of the National Nature Reserves in Anhui Province, China. For all of these strains, each gene segment was sequenced to analyse phylogenetic and speculate its origin. We also preliminarily evaluated the potential threat of each strain to mice. Our study highlights the importance of performing regular surveillance of AIV strains in Anhui Province, China as an integral part of worldwide efforts to better understand AIV ecology and prevent the emergence of novel, potentially pandemic strains. Surveillance of avian influenza virus (AIVs) in wild birds for early warning, prevention, and control of viral outbreaks should be enhanced to reduce the risk of pathogen emergence from wildlife host reservoirs.

## Methods

### Virus isolation and identification

In spring and autumn of 2014, 2970 faecal samples were collected from wild birds in Anhui Province, China. Fresh, single faecal samples were collected with cotton swabs and placed into 5 ml EP tubes with 2 ml virus protection solution (phosphate-buffered solution (pH 7.2) supplemented with penicillin, streptomycin, and 10% glycerinum). The handheld end of each cotton swab was broken off, and the remainder was placed into the sample tube and covered tightly. The handheld ends of the swabs were inserted into the ground to designate collection locations to avoid repetition in sampling.

Bird species were identified based on morphological characteristics observed by binoculars before sampling and were further determined based on faecal shape and colour in addition to DNA barcoding [20]. The samples were vortexed, oscillation and finally centrifuged, and the collected supernatant was inoculated into 9-day-old specific-pathogen-free (SPF) chicken embryos. Allantoic fluid was harvested after 72 h of culture. The HA activity of the allantoic fluid was then evaluated using an HA test. Following this, influenza virus and HA subtypes were identified using a hemagglutination inhibition (HI) test [21] with H6 mono-antiserum and further verified using subtype-specific real-time PCR (RT-PCR) [22, 23]. Virus was standardized to a concentration of 4 HAU/25 μl prior to using the HI assay to identify HA subtype. Working in a PBC, 25 μL of standardized test virus for each unknown isolate was dispensed into a series of three wells (in triplicate) in a U-bottom microtitre plate. Additionally, 25 μL of a corresponding HA-subtype positive-control antigen was dispensed at a 4 HAU/25 μL concentration into positive-control wells. Then, 25 μl of the appropriate standardized antiserum was added to the first well of the HA-subtype series. The antiserum in the antigen wells was then serially diluted beginning with the first well (25 μL carry-back with the excess 25 μL from the final row being discarded). In this way, the serum was diluted into standardized antigen. Each subtype series was diluted as soon as possible after the addition of the antiserum for that series. After this step, 25 μL of liquid remained in each well. The plate was then covered and incubated at room temperature for 25 min. Following this, 25 μL of 1% chicken erythrocyte suspension was added to each well, and the plate was submitted to gentle shaking/agitation. The erythrocyte solution was mixed periodically during this step to ensure an even suspension of erythrocytes during the dispensing process. The plate was then covered with microtitre plate-sealing tape (the PBS was removed from the plates after sealing with the tape) and incubated at room temperature until a distinct button formed in the positive-control wells, which typically required 20–30 min. The assay plates were initially observed after approximately 20 min of incubation and checked frequently thereafter for evidence of hemagglutination. Because some isolates can elute (i.e., detach from erythrocytes) in as little as 30 min, the time window for evaluation of assay results is short in some cases [21]. NA subtypes were directly analysed using subtype-specific RT-PCR and sequencing analysis. Viral RNA was extracted from the allantoic fluid samples that were positive in the HA test using TRIzol reagent. Next, cDNA was synthesized by reverse transcription with the Uni12 primer. Viral genomes were PCR-amplified using primers complementary to the conserved promoter and non-coding region of each gene segment (Table 1). The PCR reaction contained 1 μl cDNA, 1 μl forward primer and reverse primer, 5 μl 10× Taq buffer (TAKARA, Japan), 4 μl 2.5 mM dNTPs (TAKARA, Japan), 1 μl Ex Taq (TAKARA, Japan) and 37 μl RNase-free water for a final volume of 50 μl. A single PCR program was used for all primers with the following conditions: initial denaturation at 95 °C for 10 min; 30 cycles of 95 °C for 30 s, 56 °C for 30 s, and 72 °C for 1.5 min; and extension at 72 °C for 10 min, after which the reaction was stored at 4 °C. The PCR products were purified using a PCR purification kit (TianGen, China) and sequenced on an Applied Biosystems DNA analyser using specific primers.

**Table 1:** Primers in RT-PCR

Primers	Sequences
H6HAFU	CAAAAGCAGGGGAAAATGAT
H6HAFL	GTAGAAACAAGGGTGTTTTTYTCTAA
N1NAU	CAAAATGAATCCAAATCAGAAGA
N1NAL	TTTTTTGAACAAACTACTTGTCAA
N2NAU	AGCAAAAGCAGGAGTAAAAATG
N2NAL	AGTAGAAACAAGGAGTTTTTTCTAAA

### Genetic and phylogenetic analyses

Nucleotide sequences were edited using the SeqMan module of the DNASTAR package, and multiple sequence alignments were compiled using Clustal W [22–24]. Phylogenetic analyses were performed with the neighbour-joining method with maximum likelihood trees using MEGA 6.0 software [25, 26]. Bootstrap values of 1,000 were used. The phylogenetic analyses were based on the following coding sequences (nucleotides): polymerase PB2 (PB2), 1 to 2280; polymerase PB1 (PB1), 1 to 2274; polymerase PBA (PA), 1 to 2151; hemagglutinin (HA), 1 to 1701; nucleoprotein (NP), 1 to 1497; neuraminidase (NA), 1 to 1410; matrix protein 1 (M), 1 to 982; and non-structural protein 1 (NS), 1 to 844.

### Mouse studies

The 50% egg infection dose (EID_50_) was calculated according to the protocol of Reed and Muench. Six-week-old female BALB/c mice (Experimental Animal Centre of Vital River, Beijing, China) were randomly divided into four infection groups (8 mice/group) and one control group (5 mice/group). Animals in the infection groups were mildly anesthetized with dry ice and infected by intranasal inoculation with a dose of 10^6^ EID_50_ (50 μl/mouse). Three of the eight mice were randomly euthanized on day 3 post inoculation (p.i.) for titration of virus from the lungs, nasal turbinate, kidneys, spleen, and brain. The remaining five mice were monitored daily for 2 weeks for changes in body weight and mortality.

## Results

### Influenza virus isolation and subtype identification

In total, 13 H6 AIV strains were isolated from 2970 faecal samples collected from wild birds in Anhui Province, China, in 2014. The strains consisted of two NA subtypes, H6N1 (3 strains) and H6N2 (10 strains), and the host for all of these viruses was Anser fabalis (bean goose) (Table 2).

**Table 2:** Information of the 13 avian influenza viruses

No.	Virus	Abbreviation	Place	Year
1	A/*Anser fabalis*/Anhui/S20/2014(H6N2)	AH/S20	Shengjin Lake	2014
2	A/*Anser fabalis*/Anhui/S39/2014(H6N2)	AH/S39	Shengjin Lake	2014
3	A/*Anser fabalis*/Anhui/S45/2014(H6N2)	AH/S45	Shengjin Lake	2014
4	A/*Anser fabalis*/Anhui/S65/2014(H6N2)	AH/S65	Shengjin Lake	2014
5	A/*Anser fabalis*/Anhui/S104/2014(H6N2)	AH/S104	Shengjin Lake	2014
6	A/*Anser fabalis*/Anhui/S148/2014(H6N2)	AH/S148	Shengjin Lake	2014
7	A/*Anser fabalis*/Anhui/L9/2014(H6N2)	AH/L9	Caizi Lake	2014
8	A/*Anser fabalis*/Anhui/L63/2014(H6N2)	AH/L63	Caizi Lake	2014
9	A/*Anser fabalis*/Anhui/L93/2014(H6N2)	AH/L93	Caizi Lake	2014
10	A/*Anser fabalis*/Anhui/L144/2014(H6N1)	AH/L144	Caizi Lake	2014
11	A/*Anser fabalis*/Anhui/L180/2014(H6N2)	AH/L180	Caizi Lake	2014
12	A/*Anser fabalis*/Anhui/L221/2014(H6N1)	AH/L221	Caizi Lake	2014
13	A/*Anser fabalis*/Anhui/L256/2014(H6N1)	AH/L256	Caizi Lake	2014

### Phylogenetic analysis of whole genes

**Fig. 1:**
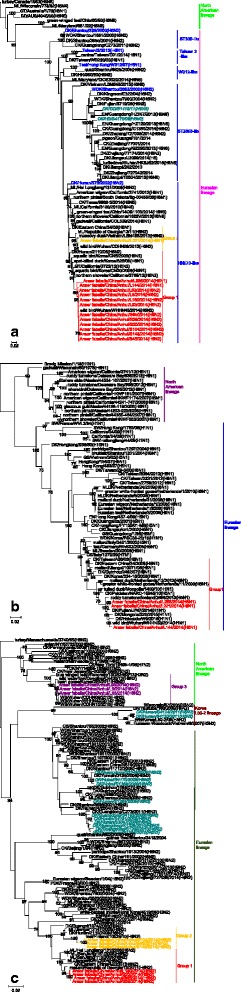
Phylogenetic analysis based on maximum likelihood of surface genes H6 (**a**), N1 (**b**) and N2 (**c**) of AIVs isolated in Anhui Province, 2014. Phylogenetic trees were generated with the MAGE 6.0 software package. The phylogenetic trees for HA (1A) were rooted to A/Turkey/Canada/1/1963 (H6N8). The phylogenetic tree for the N1 genes were rooted to A/Brevig_Mission/1/18(H1N1) and N2 genes were rooted to turkey/Massachussetts/3740/65(H6N2). The genomic sequences of the viruses listed in black and blue were downloaded from available databases; the viruses listed in red, yellow, and/or purple was sequenced in this study. The sequences shown in cyan and italics in Fig 1c were obtained from a paper by Wang GJ. Abbreviations: CK, chicken; DK, duck; GS, goose; SW, swine; ML, mallard; GT, green-winged teal; PD, Pacific black duck; WDK, wild duck; MLDK, mallard duck; WB, wild bird; EN, environment

The HA genes of all 13 H6 isolates, including 3 H6N1 viruses and 10 H6N2 viruses, belonged to the Eurasian lineage and were further divided into 2 groups according to nucleic acid homology (>95%). The homology of the 13 viral strains ranged between 93.1 and 100%. Group 1 consisted of only one virus, AH/L221, which exhibited the highest sequence identity with A/MDK/Vietnam/LBM455/2013(H6N2) in a GenBank search as well as high sequence identity with A/duck/eastern China/34/2005(H6N1) isolated from poultry. The 12 strains in group 2 exhibited high sequence identity with A/QA/Korea/CN20/2009(H6N1). Among poultry influenza viruses, these 12 viruses exhibited high sequence identity with A/duck/eastern China/11/2008(H6N1) (Fig. 1a). The three N1 NA genes formed only one group, and their homology was greater than 98% (Fig. 1b). The ten N2 NA genes exhibited greater diversity than the HA genes and formed 3 groups. Three N2 genes had a North American lineage, and seven other NA genes had a Eurasian lineage. The homology of the nucleotide sequences ranged between 86.5 and 99.8% (Fig. 1c).

**Fig. 2:**
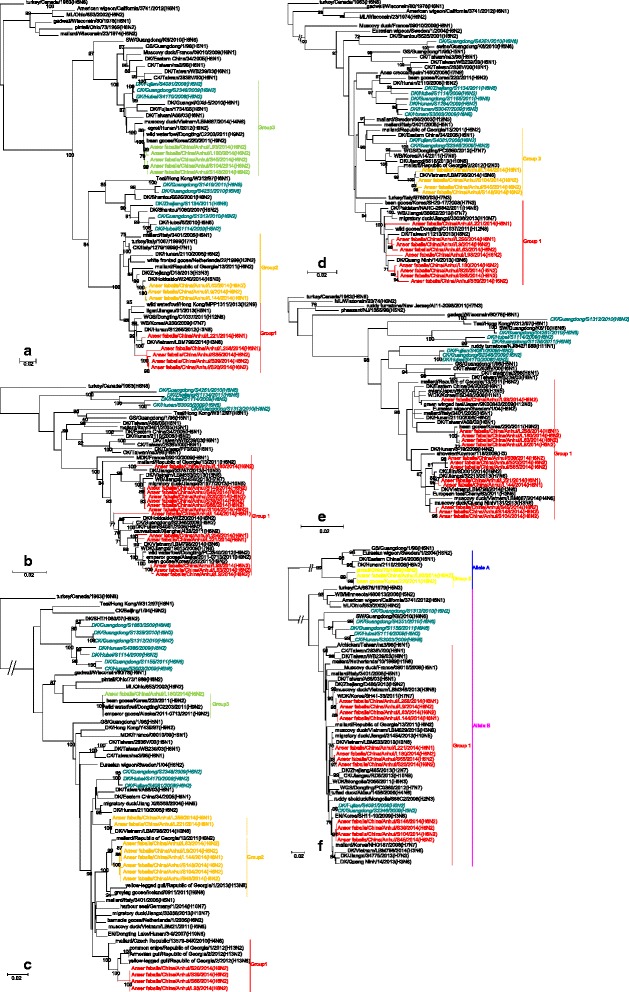
Phylogenetic analysis by maximum likelihood of inner genes of H6 subtype AIVs isolated in Anhui Province, 2014: (**a**) PB2, (**b**) PB1, (**c**) PA, (**d**) NP, (**e**) M, and (**f**) NS. The colours of the virus names listed in the PB2, PB1, PA, NP, M, and NS trees match those used in the genotype table. The phylogenetic trees of inner genes were rooted to A/Turkey/Canada/1/1963 (H6N8). The nucleotide sequences of the viruses listed in black were downloaded from GenBank; the viruses listed in red, yellow, or green were sequenced in this study. The sequences shown in cyan and italics were obtained from a paper by Wang GJ. Abbreviations: CK, chicken; DK, duck; GS, goose; SW, swine; WDK, wild duck; WB, wild bird; WGS, wild goose; EN, environment

To better understand the evolution of the H6 AIV strains in this study, 6 internal genes from the 13 viruses were phylogenetically analysed as a whole. These genes exhibited more diversity than the surface genes and were further divided into eight genotypes (Table 3). The nucleotide and amino acid homologies for the 13 H6 isolates in this study were 86.8–100% and 96.1–99.9% for the PB2 gene, 95.6–100% and 99.1–100% for the PB1 gene, 90.3–100% and 97.9–100% for the PA gene, 93.5–100% and 96.4–100% for the NP gene, 96.6–100% and 98.5–100% for the M gene, and 71.2–100% and 63.7–100% for the NS gene, respectively. All of the internal genes belong to a Eurasian lineage except for the PA gene of strain AH/L180 and the NS gene of strain AH/L93, which belong to the North American lineage. The genes were further classified into various subgroups within each lineage. The PB2 gene originated from poultry and wild birds in countries outside of China (i.e., Vietnam, Korea, and Republic of Georgia) and was divided into three groups (Fig. 2a). The PB1 gene, which showed few differences in nucleotide sequences among different strains, was classified into one group (Fig. 2b), while the PA gene was classified into three groups (Fig. 2c). The NP genes from the 13 strains were divided into two groups (Fig. 2d): three originated from ducks in Vietnam and the other ten originated from mallard in Republic of Georgia. The M genes of all 13 stains had high identity and were placed into one group (Fig. 2e). In addition, the NS phylogenetic tree showed that the NS genes of the H6 AIVs were clearly divided into two genetic lineages, termed allele A and allele B, and most of the H6 AIVs contained Eurasian allele B, although one virus from strain AH/L93 had allele A. The NS gene was classified into two groups (Fig. 2f).

**Table 3:**
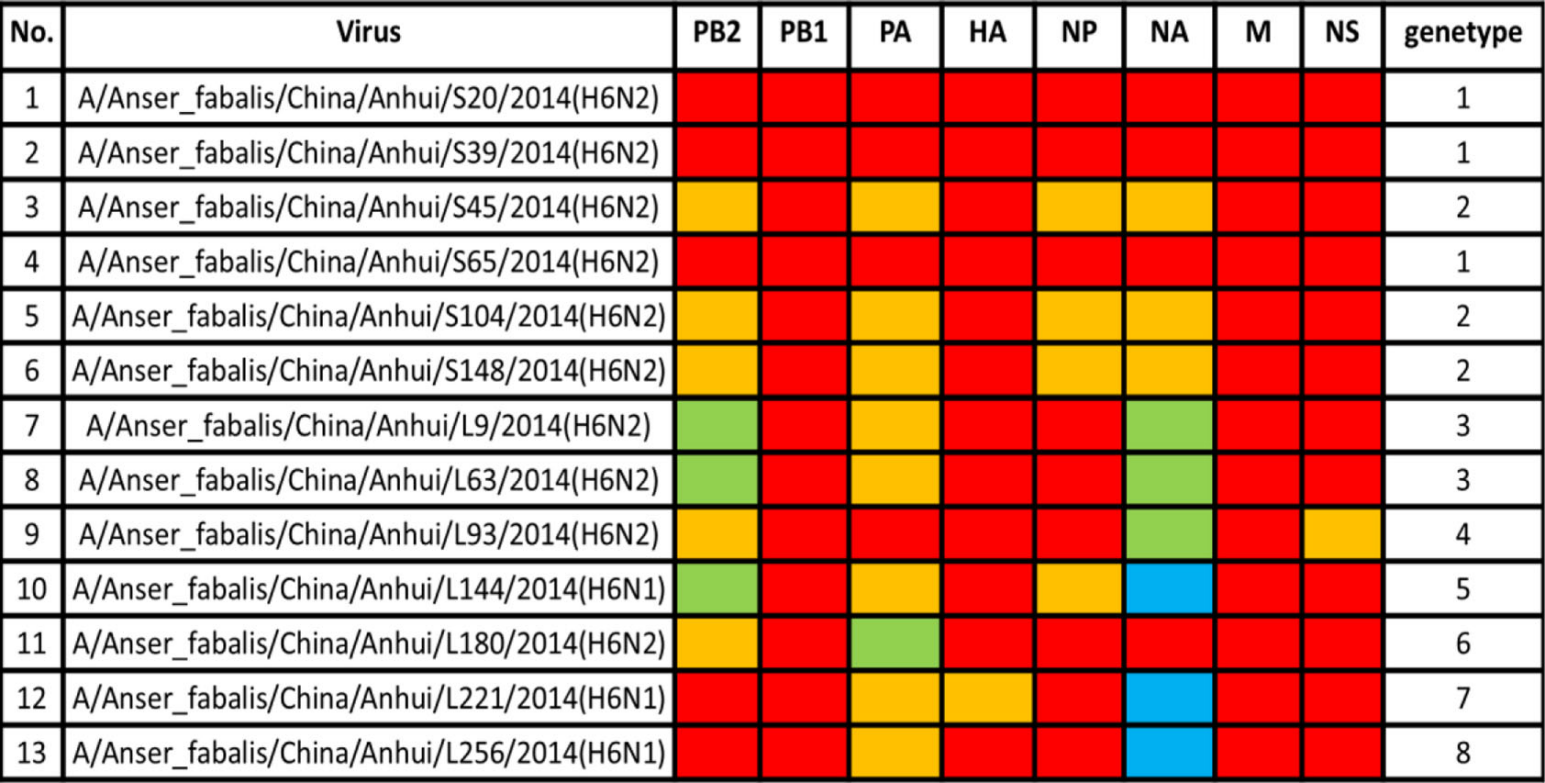
The genotypes of 13 H6 AIVs

Preliminary analyses of the possible origins of the strains were conducted. There were three Genotype 1 viruses, which were tri-reassortment of H6N2, H5N1 and H3N6. There were three Genotype 2 viruses, which were tri-reassortment of H6N2, H9N2 and H3N6. There were two Genotype 3 viruses, which were bi-reassortment of H6N2 and H9N2. There was only one virus in Genotype 4 and one in Genotype 5: both were tri-reassortment of H6N2, H9N2 and H5N1. There was only one virus in Genotype 6, which was a bi-reassortment of H6N2 and H9N2. There was only one virus in Genotype 7 and one in Genotype 8: both were tri-reassortments of H6N2, H6N1 and H3N6 (Fig. 4).

### Molecular characterization of viral genes

The isolated H6 strains, except for AH/L221, contained seven potential glycosylation sites according to analysis performed at http://www.cbs.dtu.dk/services/NetNGlyc; these included sites at positions 26 to 28, 27 to 29, 39 to 41, 306 to 308, and 311 to 313 on HA1 and at positions 498 to 500 and 557 to 559 on HA2 (H3 numbering, which is used throughout the manuscript). Strain AH/L221 contained eight potential glycosylation sites, including a glycosylation site on NNT at positions 182 to 184. All viruses except for strain AH/L144 contained the PQIETR/G motif at the cleavage site between HA1 and HA2. The cleavage site on AH/L144 contained the PLIETR/G motif, which includes an amino acid substitution from Q to L. None of the strains contained consecutive basic amino acids within the motif, and all conformed to the characteristics of low-pathogenicity AIVs. The amino acid substitutions A138S, E190V, P186L [27], Q226L, and G228S, as well as the absence of glycosylation at positions 158 to 160 in HA, have been reported to increase the affinity of influenza virus for human-type receptors [28]. The above-mentioned changes were not detected in any of the 13 H6 avian viruses sequenced. Necklace deletion in the NA gene confers enhanced virus lethality in mice. In the present study, none of the 13 H6 AIVs studied had a necklace deletion in the NA gene [29]. Some amino acid substitutions, including T271A, E627K, and D701N in PB2, contribute to increased virulence and transmission of influenza viruses in mammals. The H6 viruses sequenced in this study did not contain any of these changes either. No Y436H substitutions in the PB1 protein or T515A substitutions in the PA protein were found, which suggests that the studied viruses have low pathogenicity for mammalian and avian hosts. Additionally, no amino acid substitutions were found in the M2 transmembrane domain, suggesting that the viruses are sensitive to M2 ion channel inhibitors [30].

The N66S substitution in the PB1-F2 protein, which has been associated with the increased virulence of the 1918 pandemic virus and the high pathogenicity of the AI H5N1 virus in mice and ferrets [31, 32], was not found in any of the H6 strains examined in this study. Additionally, none of the viruses harboured the S31N substitution in the M2 protein, indicating that all are sensitive to amantadine inhibitors [33]. The virulence of influenza virus in humans is related to resistance to the antiviral effects of cytokines, such as interferon (IFN). In particular, the D92E mutation in the NS1 protein increases resistance to IFN; this mutation was not observed in any of the strains evaluated in this study [34].

### Replication of H6 viruses in mice

**Fig. 3:**
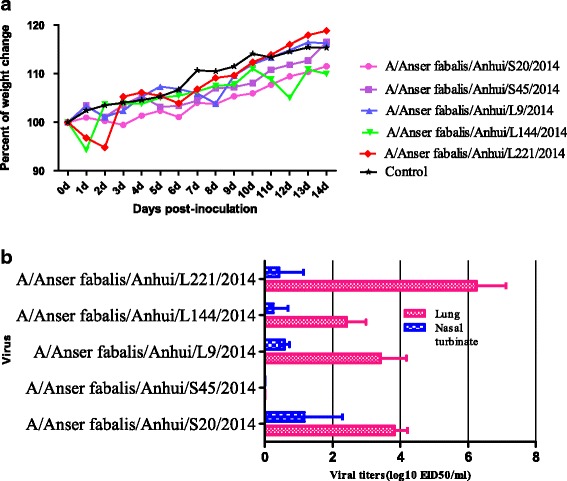
Changes in body weight. Body weight (**a**) and mortality (**b**) in BALB/c mice inoculated with strains A and B of H6 viruses. **a** Body weights and survival rates of mice were observed over 14 days after infection. **b** Lungs and trachea were collected at 3 days post infection (p.i.), and virus replication levels were measured by EID_50_ values in specific-pathogen-free (SPF) eggs

To investigate the replication ability and virulence of the isolated H6 strains in mammals [35], five H6 strains were selected according to their NA gene subtype and phylogenetic characteristics of HA and NA and used to infect mice. Following infection, none of the viruses were detected in the spleen, kidneys, or brain of the mice. Four of the five viruses caused the mice to lose weight (Fig. 3a). In virus proliferation tests in chicken embryos, one strain of virus, strain AH/S45 (H6N2), was not detectable in any organs, whereas the four other strains were detected in the lungs (mean titre 2.4 to 6.3 log10 EID_50_) and nasal turbinate (mean titre 0.6 to 1.3 log10 EID_50_) (Fig. 3b). These results indicate that the majority of H6 influenza viruses that were isolated from wild birds can replicate in the respiratory systems of mice without preadaptation.

## Discussion

Based on body shape, physiological characteristics, and other features, wild birds have unique flight patterns, which has led to their wide geographical spread, strong regional activity and extended seasonal migration. Wild birds migrate from breeding to wintering places each year along unique migration routes. Wild aquatic birds are the natural host of influenza virus and can act as a reservoir for the transfer of genes between species, forming transient “genome constellations” that are continually reshuffled by reassortment [36, 37]. The birds’ migration behaviours expand the distribution of the virus and provide conditions for the cross-border spread of viruses. Many reports have analysed the biological and pathogenic characteristics of influenza virus in poultry in China; however, studies of influenza virus in wild birds are relatively rare.

In recent years, several AIV subtypes have been circulating and evolving in southern China; these include the H5, H9, and H6 subtypes as well as the newly emerged H7N9 and H10N8 viruses. This has led to frequent avian influenza outbreaks in poultry and humans. Anhui Province is located in south-eastern China (29°41′to 34°38′N, 114°54′to 119°37′E). Lying in the middle and lower valleys of the Yangtze and HuaiHe rivers, Anhui is abundant in wetlands, with wetland areas accounting for 21% of the total area of the province. These areas provide important stopover and wintering grounds for birds migrating along the East Asia-Australia migratory flyway. In the fall and winter of each year, a large number of migratory birds gather in the wetlands, resulting in extremely high population densities of the same or different species, which likely favours the spread and viral gene reassortment of the influenza virus. As an important agricultural province, Anhui is also home to a great number of poultry raised under different farming practices. These poultry often come into close contact with wild birds, creating the opportunity for cross-species AIV transmission.

In 2 years of surveillance work in Anhui, we obtained 31 AIV strains from 2970 faecal samples. Among these strains, eight subtype combinations, including H1N1, H1N2, H3N3, H3N8, H6N1, H6N2, H9N2, and H11N9, were identified using HA-HI tests and PCR. The H6 subtype accounted for the largest proportion of those identified, being found in approximately 41.9% (13/31) of the samples. H6N2 accounted for the majority of these H6 AIVs (10/13). Overall, the goal of this work was to obtain baseline information regarding the epidemiological and virological characteristics of H6 virus strains that exist in wild waterfowl. Toward this aim, we assessed 13 strains of H6 virus isolated from faecal samples of bean goose (Anser fabalis) in Anhui Province, China in 2014.

**Fig. 4:**
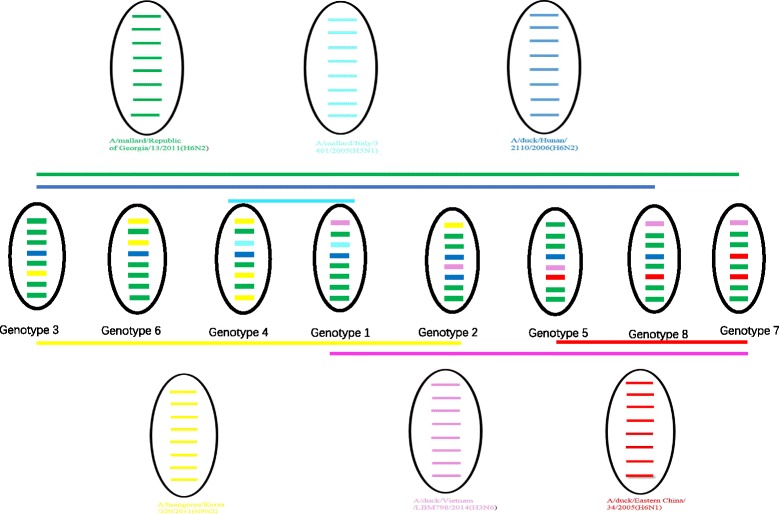
A simplified schematic showing the putative genomic composition of the novel H6N1 and H6N2 reassortant avian influenza viruses identified in this study. The eight gene segments (from *top* to *bottom*) in each virus are PB2, PB1, PA, HA, NP, NA, M, and NS. Each colour represents a separate source background. Eight genotypes are listed here. The six different colours represent separate virus backgrounds. The illustration is based on nucleotide-distance comparisons and phylogenetic analyses

These 13 H6 strains originated from complicated reassortment between viruses found in poultry and wild birds from China and other countries (Fig. 4). Interestingly, all of the HA genes originated from poultry in China. Except for strain AH/L221, which originated from the A/duck/Eastern China/34/2005(H6N1)-like gene pool, the other 12 H6 strains all originated from the A/duck/Hunan/2110/2006(H6N2)-like gene pool. The ten N2 strains all exhibited high sequence identity with wild birds from different countries and were reassortment between H9N2 and H6N2 in bean goose, duck, and mallard. Three N1 genes were found in H6N1 isolates from poultry in their native countries. The internal genes also showed complicated reassortment from varied sources, including poultry and different wild bird species from China and other countries. This finding further shows that wild birds, as the natural host of AIV, play an important role in gene reassortment or rearrangement of viruses, which can subsequently be transmitted to domestic poultry. A former study showed that the gene flow and reassortment of the Eurasian lineage in the North American gene pool dramatically changed the evolutionary dynamics of influenza virus in natural reservoir hosts [38]. The HA, PA and NS genes in the strains examined in this study had high identity with A/bean goose/Korea/220/2011(H9N2), which belongs to the North American lineage [38]. China and Korea are both along the same bird migration pathway, which increases the gene flux of influenza virus between the two countries and may accelerate evolution driven by North American-Eurasian reassortment. During seasonal migration for breeding and wintering, migratory birds can distribute genes to local poultry or wild birds and can acquire additional genes [39]. This invasion of viruses from wild bird reservoirs can increase the risk of emergence of highly pathogenic avian influenza (HPAI) viruses and threaten food security in addition to increasing the chance of virus infection in humans.

Gillim-Ross et al. [40] described 14 strains of H6 virus from poultry that could infect and replicate in the lungs of mice and showed that some of these strains are lethal. Wang et al. also reported that 37 of 38 tested H6 viruses from poultry could replicate efficiently in the lungs of mice [17]; however, none were lethal. In the present study, experimental infection in mice revealed that all but one of the studied H6 strains could replicate in the lungs and turbinate of mice without adaptation. Some of the viruses also induced weight loss in mice. These data support the idea that H6 AIVs from wild birds can replicate in BALB/c mice without adaptation and that mammals can be directly infected with wild-bird AIV. Following infection, the AH/S45 strain was not found in any organs, but this strain has high sequence identity with strain AH/S20: these two strains have no reported differences in amino acid sequence. Further research is required to determine whether differences in amino acids that influence the viral replication ability exist between these two viruses. Some studies have reported that red blood cells from turkeys, sheep, and horses possess a-2, 3 receptors; however, red blood cells in humans with Type O blood and those from guinea pigs possess a-2, 6 receptors. Interestingly, in the present study, the 13 H6 AIV strains studied all exhibited high HA activity in red blood cells from horses and sheep but low activity in red blood cells from Type O humans and from guinea pigs. These results coincide with the properties bestowed by amino acids 226 and 228 in the HA gene of the virus, which together form the characteristic poultry receptor-binding site on the virus [27].

H6 AIV has a worldwide distribution, similar to H5N1 and H9N2 [41, 42], and has caused considerable losses in poultry farming. In one report, out of a total of 15,689 serum specimens gathered from 22 provinces in mainland China, approximately 63 specimens were positive for H6 AIV [12]. These cases of H6-infected humans serve as a warning. As the natural host of influenza virus, wild birds, especially migratory species, play an indispensable role in the spread and reassortment of AIV and provide unknown opportunities for the mutation and emergence of novel influenza viruses [43]. To fully understand the ecology, biology, and potential hazards of influenza virus, regular surveillance of all influenza virus hosts, and especially wild birds and poultry, is necessary. The data generated from such efforts must be combined to accurately assess the potential threat. Notably, the present study provides only a general overview of the H6 virus strains that are currently circulating among Anseriformes in Anhui, China. More intensive sampling and sequencing of AIVs from wild birds is needed to help refine our understanding of host specificity and viral evolution [44], which will also reveal the processes driving viral adaptation and maintenance in alternative hosts.

## Conclusion

The H5, H6, and H9 subtypes of influenza A virus are found worldwide. Although it is known that H6 AIV strains found in poultry pose a potential threat to humans, few studies have surveyed this subtype in infected wild birds, and its threat to humans is therefore not well understood. In the present study, we performed a risk assessment of H6 influenza A virus transmission using phylogenetic and pathogenic analyses to better understand the potential threat of wild birds, especially migratory birds, transmitting influenza virus to poultry and/or humans. In doing so, we identified 13 strains of H6 AIV in Anseriformes. Some of these strains were reassortment from the Eurasian and North American lineages. Overall, we conclude that H6 AIV strains isolated from wild birds have the potential to infect mammals and humans.

## Abbreviations

AIV: Influenza A virus
HA: Hemagglutinin
M: Matrix
NA: Neuraminidase
NP: Nucleoprotein
NS: Non-structural
PA: Acidic polymerase
PB1: Polymerase basic 1
PB2: Polymerase basic 2
RT-PCR: Reverse-transcription polymerase chain reaction
